# Remote Assessment of Disease and Relapse in Major Depressive Disorder (RADAR-MDD): Recruitment, retention, and data availability in a longitudinal remote measurement study

**DOI:** 10.1192/j.eurpsy.2022.315

**Published:** 2022-09-01

**Authors:** F. Matcham, D. Leightley, S. Siddi, F. Lamers, K. White, P. Annas, G. De Girolamo, S. Difrancesco, J.M. Haro, M. Horsfall, A. Ivan, G. Lavelle, Q. Li, F. Lombardini, D. Mohr, V. Narayan, C. Oetzmann, B. Penninx, S. Simblett, S. Bruce, R. Nica, T. Wykes, J. Brasen, I. Myin-Germeys, A. Rintala, P. Conde, R. Dobson, A. Folarin, C. Stewart, Y. Ranjan, Z. Rashid, N. Cummins, N. Manyakov, S. Vairavan, M. Hotopf

**Affiliations:** 1 Institute of Psychiatry, Psychology & Neuroscience, King’s College London, Department Of Psychological Medicine, London, United Kingdom; 2 Institute of Psychiatry, Psychology & Neuroscience,King’s College London, Department Of Psychological Medicine, London, United Kingdom; 3 Parc Sanitari Sant Joan de Déu, Fundació Sant Joan De Déu, Cibersam, Barcelona, Spain; 4 Department of Psychiatry and Amsterdam Public Health Research Institute, Amsterdam Umc, Amsterdam, Netherlands; 5 H. Lundbeck A/S, N/a, Valby, Denmark; 6 IRCCS Instituto Centro San Giovanni di Dio Fatebenefratelli, N/a, Brescia, Italy; 7 Janssen Research and Development, LLC, N/a, New Jersey, United States of America; 8 Center for Behavioral Intervention Technologies, Department Of Preventative Medicine, Northwestern University, United States of America; 9 Institute of Psychiatry, Psychology & Neuroscience,King’s College London, Psychology, London, United Kingdom; 10 King’s College London, Institute Of Psychiatry, Psychology And Neuroscience, London, United Kingdom; 11 KU Leuven, Neurosciences, Leuven, Belgium; 12 Janssen Pharmaceutica NV, N/a, Beerse, Belgium

**Keywords:** remote measurement technologies, longitudinal, major depressive disorder, observational

## Abstract

**Introduction:**

Major Depressive Disorder (MDD) is prevalent, often chronic, and requires ongoing monitoring of symptoms to track response to treatment and identify early indicators of relapse. Remote Measurement Technologies (RMT) provide an exciting opportunity to transform the measurement and management of MDD, via data collected from inbuilt smartphone sensors and wearable devices alongside app-based questionnaires and tasks.

**Objectives:**

To describe the amount of data collected during a multimodal longitudinal RMT study, in an MDD population.

**Methods:**

RADAR-MDD is a multi-centre, prospective observational cohort study. People with a history of MDD were provided with a wrist-worn wearable, and several apps designed to: a) collect data from smartphone sensors; and b) deliver questionnaires, speech tasks and cognitive assessments and followed-up for a maximum of 2 years.

**Results:**

A total of 623 individuals with a history of MDD were enrolled in the study with 80% completion rates for primary outcome assessments across all timepoints. 79.8% of people participated for the maximum amount of time available and 20.2% withdrew prematurely. Data availability across all RMT data types varied depending on the source of data and the participant-burden for each data type. We found no evidence of an association between the severity of depression symptoms at baseline and the availability of data. 110 participants had > 50% data available across all data types, and thus able to contribute to multiparametric analyses.

**Conclusions:**

RADAR-MDD is the largest multimodal RMT study in the field of mental health. Here, we have shown that collecting RMT data from a clinical population is feasible.

**Disclosure:**

No significant relationships.

